# Effectiveness of Balance Rehabilitation Unit (BRU) Posturography Versus Conventional Rehabilitation in Patients With Unilateral Peripheral Vestibular Dysfunction

**DOI:** 10.7759/cureus.46217

**Published:** 2023-09-29

**Authors:** Ana Teresa de la O-Gómez, Jose Alfredo Sierra-Ramírez, Daniel Ramos-Maldonado, Marta Elena Hernández-Caballero

**Affiliations:** 1 Servicio de Otoneurología, Instituto Nacional de Rehabilitación Luis Guillermo Ibarra Ibarra, Ciudad de Mexico, MEX; 2 Sección de Estudios de Posgrado e Investigación, Escuela Superior de Medicina, Instituto Politécnico Nacional, Ciudad de Mexico, MEX; 3 Servicio de Otoneurología, Instituto Nacional de Rehabilitación Luis Guillermo Ibarra Ibarra, Ciudad de Mexico, MEX; 4 Biomedicina, Facultad de Medicina, Benemérita Universidad Autónoma de Puebla, Puebla, MEX

**Keywords:** balance, virtual reality, upvd, conventional rehabilitation, posturographic balance rehabilitation unit

## Abstract

Background: Patients with unilateral peripheral vestibular deficit (UPVD) experience vertigo, dizziness, disability, negative influences on their quality of life, anxiety, and depression. In vestibular rehabilitation, virtual reality (VR) has proven to be effective. This investigation sought to evaluate the efficacy of the Balance Rehabilitation Unit (BRU^TM^) (Medicaa^TM^ Montevideo, Uruguay, Balance Suite, version BRU 415) in patients with UPVD.

Methods: A prospective, randomized, controlled study involved 38 patients from the Otoneurologic Service at the National Institute of Rehabilitation "Luis Guillermo Ibarra Ibarra" in Mexico. A physician specialist diagnosed the patients with UPVD and assigned them randomly to one of two groups. Group 1 (n = 19) received traditional vestibular rehabilitation, whereas Group 2 (n = 19) received BRU^TM^-supported vestibular rehabilitation. Both groups were monitored by medical professionals. Patients were evaluated with the Dizziness Handicap Inventory, static and dynamic balance assessments, the dynamic gait index, and the sensory organization test. The statistical analysis was conducted using the Student's t-test, with p 0.05 considered statistically significant.

Results: The difference in mean age between the conventional therapy and BRU^TM^ groups was not statistically significant. Both conventional vestibular rehabilitation and the BRU^TM^ led to statistically significant improvements in all assessed parameters, with no statistically significant differences between the two groups.

Conclusion: Balance, mobility, and quality of life were enhanced similarly in UPVD patients by BRU^TM^-supported vestibular rehabilitation and conventional vestibular rehabilitation. In addition, BRU^TM^ facilitated patient motivation, exercise feedback, and confidence enhancement.

## Introduction

Unilateral impairment of the peripheral vestibular sensory organs or vestibular nerves is known as unilateral peripheral vestibular dysfunction (UPVD), and it can be caused by a variety of factors, including disease, infection, trauma, toxicity, genetics, neurodegeneration, and postoperative factors. In approximately 50% of cases, however, the etiology remains unknown [1]. Agrawal et al. (2009) estimate that 35.4% of adults require medical treatment for vestibular dysfunction [2]. With age, vestibular function tends to decline. Grill et al. (2018) indicate that peripheral vestibular dysfunction is more common in the elderly (32.1%) than in younger and middle-aged persons (2.4%), and adolescent vestibular impairment has been estimated to occur in 0.45% of the population [3,4].

Clinical manifestations of vestibular dysfunction include symptoms such as dizziness, nausea, vertigo, visual disturbances, spatial disorientation, gait instability, and balance impairment. These symptoms are frequently chronic and debilitating, which makes differential diagnosis difficult [1]. Inadequate compensation for vestibular dysfunction severely restricts a person's ability to engage in daily activities like transportation, working, and exercising. These restrictions can cause anxiety, melancholy, and a decrease in life quality. In addition, the socioeconomic burden of work-related disabilities is substantial [5]. In addition to affecting spatial navigation, vestibular dysfunction can impair memory, executive function, and attention [6].

Individuals with chronic vestibular dysfunction and UPVD have been shown to benefit significantly from vestibular rehabilitation exercises. Vertigo may be alleviated, postural stability can be improved, and visual acuity can be increased during head movements with the use of these exercises [1,5,7]. Rehabilitation approaches have to center around making up for vestibular input deficiencies during the gait cycle [8]. Nevertheless, in conventional rehabilitation, the lack of medical supervision during exercise performance makes it difficult for patients to receive feedback, resulting in repetitive and monotonous exercises that may lead to therapy discontinuation [9].

Patients with vestibular disorders have been the focus of previous studies looking into the efficacy of virtual reality for balance training, and the incorporation of technology and self-learning methods into vestibular rehabilitation may offer an alternative for individuals with UPVD [10-12]. The Balance Rehabilitation Unit (BRU^TM^) system employs visual stimuli to detect abnormalities in postural control in patients with benign paroxysmal positional vertigo [13], and balance velocity abnormalities in patients with Meniere's disease [14]. However, information on the BRU^TM^ system's effectiveness in vestibular therapy for those with UPVD is scarce. Therefore, the aim of this research was to compare traditional vestibular therapy with the BRU^TM^ system for treating UPVD patients and determine which approach was more effective.

## Materials and methods

In the Otoneurologic Department of Mexico's National Institute of Rehabilitation, "Luis Guillermo Ibarra Ibarra" conducted this prospective, randomized, single-center, controlled study. The Institutional Research Ethics Committee approved Protocol #07/16. Furthermore, the study followed the guidelines set forth by the Helsinki Declaration.

Participants included men and women aged 30-60 who were diagnosed with UPVD by a physician specialist using a combination of patient history, physical exam, and vestibular and audiological testing. Patients with cognitive impairments, mobility limitations due to musculoskeletal or systemic diseases, balance limitations due to neurological disorders, vision impairments, or a known medical history of psychiatric or neurological conditions that could impair patient cooperation or cognitive function were not included in the study. Informed consent was given by all individuals.

Randomization and assignment to groups

Patients were randomly assigned to the control (n = 19) or experimental (n = 19) groups. The control group received conventional vestibular rehabilitation, while the experimental group received vestibular rehabilitation with the BRU^TM^ (Medicaa^TM^ Montevideo, Uruguay, Balance Suite, version BRU 415) system.

Conventional vestibular rehabilitation

In the control group, patients were treated with the standard vestibular rehabilitation program under the supervision of a physician specialist. Following the exercises originally described by Cooksey and Cawthorne [15] and adding more specific components for vestibular rehabilitation [1]. The goal of the therapy was to improve vestibular function by stimulating the central nervous system to make up for the damage. Five days in a row, the patients participated in a 30-minute training session involving exercises performed in a hierarchical order of difficulty, beginning with eye movements with the head immobile, then eye and head movements, then arm and body movements, and concluding with exercises that required standing up and movement.

Vestibular rehabilitation with BRU^TM^

Patients in the experimental group received vestibular rehabilitation under the supervision of a physician specialist using the Balance Rehabilitation Unit (BRU^TM^) system. The BRU^TM^ setup included a metal framework, a harness and protective belt, a platform, virtual reality goggles, a computer with a keyboard and mouse, and licensed software (Medicaa^TM^ Montevideo, Uruguay, Balance Suite, version BRU 415). Five days in a row, the patients were required to stand on the platform for 30 minutes during a training session. Included in the exercises were saccadic stimuli, delayed tracking, optokinetic stimuli, and visual-vestibular integration. Furthermore, the study included several sensory stimulation modifications and an escalation in task difficulty. The study involved the investigation of different optokinetic stimuli, which encompassed slow tracking, optokinetic stimuli with progressively increasing speeds, an optokinetic tunnel, a linear optokinetic train lacking panorama, a circular optokinetic train lacking panorama, a linear optokinetic train with panorama, and a circular optokinetic train with panorama. The investigation conducted in this research centered on the integration of visual and vestibular information, with a special emphasis on horizontal cephalic motions [16].

Outcome measures

The patients' balance and perceived disability were evaluated using a variety of objective and subjective measures [17]. Using the validated Spanish version of the Dizziness Handicap Inventory (DHI), which consisted of 25 items organized into three content domains, the functional, affective, and physical impact of vertigo was assessed; 0 to 30 for mild, 31 to 60 for moderate, and 61 to 100 for severe. The rate of change between pre- and post-DHI scores was defined as the vestibular rehabilitation outcome [18,19].

Dysfunction in the vestibular system was evaluated by applying static and dynamic balance tests; patients were screened for balance using the standing balance test (Romberg) [20], and the walking balance test (tandem walking) [21], for spatial orientation using the Fukuda stepping test [22], and by the analysis of gait, deviation using the Babinski-Weill or star gait. The results were determined by the ratio of modified to unmodified responses between the pre- and post-tests.

The dynamic gait index (DGI) [23] was used to assess fall risk in patients with UPVD. This index measures dynamic balance during gait and consists of eight items with values ranging from 0 to 3 for a possible 24; when scores were less than 19 points, the chance of falling increased.

Balance parameters on the sensory organization test (SOT) were used to calculate the significance of somatosensory, visual, and vestibular input to overall postural stability [24] on a computerized dynamic posturography system (Neurocom® Smart Balance Master; Neurocom International, Inc., Clackamas, Oregon), with measurements performed in triplicate and scores that ranged from 0 to 100% on six sensorimotor conditions and a composite score.

Statistical evaluation

As appropriate, data were presented as group means and standard deviation (SD), or numbers (percentages). Continuous variables were tested for normality using the Kolmogorov-Smirnoff method. The Student's t-test and the nonparametric Mann-Whitney U-test were utilized to compare continuous and nominal variables, respectively. The statistical analysis was conducted using SPSS software, version 23.0 (IBM Corp., Armonk, NY) for Windows, with a significance level of p 0.05.

## Results

A total of 38 patients with a diagnosis of unilateral peripheral vestibular dysfunction who met the inclusion criteria were randomly assigned to the following groups: 19 patients in the conventional rehabilitation group had a mean age of 46.7 ± 9.3 years, with 10 female patients (53%) and nine male patients (47%), whereas 19 patients in the BRU^TM^ therapy group had a mean age of 44.0 ± 10.3 years, with 13 female patients (66%) and six male patients (34%). Regarding age and gender, there were no significant differences (Table 1).

**Table 1:** Characteristics of patients (n = 38) BRU: Balance Rehabilitation Unit. *Student's t-test, **Mann–Whitney U test, Level of significance p < 0.05.

		Conventional Rehabilitation	BRU	p
n		19	19	
Age (years) Media ± SD		46.7 ± 9.3	44.0 ± 10.4	0.40*
Gender	Male, n (%)	9 (47)	6 (34)	0.32**
	Female, n (%)	10 (53)	13 (66)	

The results of the DHI's assessment of the emotional, physical, and functional impact of dizziness on the patient are presented in Table 2. After receiving specific rehabilitation (conventional or BRU^TM^), one observed a decrease in the degree of disability in all areas (emotional, physical, and functional) in both groups; the levels of disability changed from severe disability to mild disability, with no statistically significant differences between therapies.

**Table 2:** Comparison of DHI scores between pretreatment and post-treatment groups in the functional, emotional, and physical areas. DHI: Dizziness Handicap Inventory); BRU: Balance Rehabilitation Unit. *Mann–Whitney U test, Level of significance p < 0.05.

		Conventional Rehabilitation n (%)	BRU n (%)	P*
DHI Functional				
Pretreatment	mild disability	10 (52.7)	9 (47.4)	
	moderate disability	4 (21.0)	4 (21.0)	0.71
	severe disability	5 (26.3)	6 (31.6)	
Post-treatment	mild disability	15 (79.0)	16 (84.3)	
	moderate disability	3 (15.7)	3 (15.7)	0.63
	severe disability	1 (5.3)	0 (0.0)	
DHI Emotional				
Pretreatment	mild disability	9 (47.4)	8 (42.1)	
	moderate disability	4 (21.0)	7 (36.9)	0.91
	severe disability	6 (31.6)	4 (21.0)	
Post-treatment	mild disability	18 (94.7)	15 (79.0)	
	moderate disability	0 (0.0)	4 (21.0)	0.10
	severe disability	1 (5.3)	0 (0.0)	
DHI Physical				
Pretreatment	mild disability	5 (26.3)	5 (26.3)	
	moderate disability	9 (47.4)	5 (26.3)	0.38
	severe disability	5 (26.3)	9 (47.4)	
Post-treatment	mild disability	15 (79.0)	13 (68.4)	
	moderate disability	2 (10.5)	6 (31.6)	0.62
	severe disability	2 (10.5)	0 (0.0)	

In the initial evaluation of patients with UPVD before receiving specific therapies, the vestibular tests to assess dynamic and static balance were altered (Table 3), whereas the tandem gait and Romberg balance tests were unaffected by conventional and BRU^TM^ rehabilitation therapies after therapy, and the Fukuda step test (conventional therapy 15.8% vs. BRU^TM^ 21.1%) and the Babinski Weill test (conventional therapy 26.3% vs. BRU^TM^ 15.4%) changed in a modest proportion of patients in both treatment groups. We found no statistically significant differences between the two rehabilitation treatments based on dynamic and static vestibular assessments of balance.

**Table 3:** Comparison of static and dynamic vestibular tests (Romberg, Tandem, Fukuda, and Babinski Weill) to assess balance exhibited alterations between pretreatment and post-treatment groups. BRU: Balance Rehabilitation Unit. *Mann–Whitney U test, Level of significance p< 0.05.

		Conventional Rehabilitation n (%)	BRU n (%)	P*
Romberg				
Pretreatment	altered	8 (42.1)	9 (47.4)	0.75
	unaltered	11 (57.9)	10 (52.6)	
Post-treatment	altered	0 (0.0)	0 (0.0)	1.00
	unaltered	19 (100.0)	19 (100.0)	
Tandem				
Pretreatment	altered	6 (31.2)	6 (31.2)	1.00
	unaltered	13 (68.4)	13 (68.4)	
Post-treatment	altered	0 (0.0)	0 (0.0)	1.00
	unaltered	19 (100.0)	19 (100.0)	
Fukuda				
Pretreatment	altered	14 (73.7)	17 (89.5)	0.22
	unaltered	5 (26.3)	2 (10.5)	
Post-treatment	altered	3 (15.8)	4 (21.1)	0.68
	unaltered	16 (84.2)	15 (78.9)	
Babinski Weill				
Pretreatment	altered	16 (84.2)	15 (78.9)	0.68
	unaltered	3 (15.8)	4 (21.1)	
Post-treatment	altered	5 (26.3)	3 (15.8)	0.43
	unaltered	14 (73.7)	16 (84.2)	

Table 4 displays the Dynamic Gait Index (DGI), a measure of dynamic balance during walking; for both groups, there is a risk of falling (conventional rehabilitation 21.1% vs BRU^TM^ 26.3%). However, following treatment, all patients in both groups were no longer at risk of falling, and there were no statistically significant differences between therapies.

**Table 4:** Comparison of Dynamic gait index between pretreatment and post-treatment groups BRU: Balance Rehabilitation Unit. *Mann–Whitney U test, Level of significance p< 0.05.

Dynamic gait index	Conventional Rehabilitation n (%)	BRU n (%)	P*
Pretreatment			
Fall risk	4 (21.1)	5 (26.3)	0.71
Unfall risk	15 (78.9)	14 (73.7)	
Post-treatment			
Fall risk	0 (0.0)	0 (0.0)	1.00
Unfall risk	19 (100.0)	19 (100.0)	

Balance evaluations using the Sensory Organization Test (SOT) revealed improvement in all balance conditions (SOT1 through SOT6 and composite SOT) following both conventional and BRU^TM^ therapies. In terms of the degree of improvement in balance, there were no significant differences between conventional therapy and BRU^TM^ (Table 5). Patients with UPVD benefited from both conventional vestibular rehabilitation and BRU^TM^ therapy in terms of reduced disability, improved balance, and the elimination of the risk of falling. In terms of the outcomes tracked in this study, both interventions demonstrated comparable efficacy.

**Table 5:** Comparison of SOT 1 to SOT 6 and SOT composite between pretreatment and post-treatment groups SOT: Sensory organization test; BRU: Balance Rehabilitation Unit. *Student's t-test, Level of significance p< 0.05.

	Conventional Rehabilitation Media (SD)	BRU Media (SD)	P*
SOT 1			
Pretreatment	92.9 ± 3.7	92.3 ± 4.4	0.541
Post-treatment	93.2 ± 2.3	93.6 ± 2.2	0.403
SOT 2			
Pretreatment	88.9 ± 6.5	89.7 ± 4.8	0.725
Post-treatment	86.7 ± 20.6	87.6 ± 19.1	0.779
SOT 3			
Pretreatment	86.3 ± 7.0	90.1 ± 4.0	0.059
Post-treatment	90.7 ± 2.9	92.4 ± 2.7	0.067
SOT 4			
Pretreatment	62.8 ± 12.0	66.3 ± 17.1	0.174
Post-treatment	75.9 ± 6.1	76.6 ± 9.0	0.529
SOT 5			
Pretreatment	37.7 ± 17.8	43.7 ± 20.4	0.328
Post-treatment	54.6 ± 15.3	55.8 ± 19.7	0.373
SOT 6			
Pretreatment	33.0 ± 20.0	44.1 ± 20.5	0.174
Post-treatment	53.6 ± 12.5	57.3 ± 21.6	0.165
SOT composite			
Pretreatment	60.6 ± 9.2	65.1 ± 11.8	0.183
Post-treatment	72.7 ± 6.8	73.5 ± 10.8	0.396

## Discussion

Treatment of peripheral vestibular problems with traditional vestibular therapy is generally accepted as a safe and effective option, with balance, daily activities, vision, and other symptoms, including vertigo and anxiety, significantly improving. As a result, it is considered the cornerstone of treatment for UPVD. Conventional rehabilitation aims to enhance the somatosensory systems, including visual and proprioceptive inputs, and promote their integration at the central nervous system level [1,25]. However, conventional vestibular rehabilitation has several limitations, including the need for physician supervision, access and time commitment issues, incorrect execution of exercises, patient engagement, and the need for active effort. One promising method of resolving these issues is the use of virtual reality (VR) in vestibular rehabilitation [16,26].

According to Heffernan et al. (2021) [27] and Viziano et al. (2019) [28], improvements in habituation, substitution, and adaptability can be achieved through more motivated vestibular rehabilitation when VR is used to treat peripheral vestibular diseases. In the current study, the efficacy of the BRU^TM^ system in treating UPVD was evaluated, and improvements were noted in several parameters, including vestibular tests for static and dynamic balance, the DHI, the DGI, and the SOT. It was determined that the BRU^TM^ system's outcomes were comparable to those of conventional rehabilitation. In addition, patient satisfaction was significantly greater among those who received VR therapy, highlighting the advantages of this modality [5,26]. Prior research likewise indicates that VR devices do not exhibit superior performance in comparison to conventional rehabilitation [11,12].

On the other hand, other studies have demonstrated that the BRU^TM^ system is more beneficial to conventional vestibular rehabilitation for improving balance outcomes in patients with Meniere's disease [14] and benign paroxysmal positional vertigo [13]. In addition, other immersive VR devices have been shown to enhance balance in peripheral vestibular dysfunction compared to conventional exercises alone [28,29]. Differences in study outcomes regarding the utility of VR devices in peripheral vestibular disorders may be attributable to differences in VR technology, exercise dosage, or outcome measures for evaluating balance maintenance [5]. Recommendations for VR-based treatment include the use of validated assessment instruments, explicit documentation of therapy duration and frequency, the interval between sessions, documentation of VR-related adverse effects and complications, and, if possible, the cost per session [16].

This study contributes novel evidence regarding the effectiveness of the BRU^TM^ system in UPVD patient rehabilitation. While the results did not demonstrate additional benefits of the BRUTM system over conventional rehabilitation, this device offers a more enjoyable method of balance training, which may improve exercise adherence and equilibrium outcomes. It is essential to consider the influential role of physician supervision in both conventional and BRU^TM^ therapies, as it was a consistent factor in the current study and other investigations [10]. The BRU^TM^ system should be considered an important tool for the rehabilitation of patients with balance disorders, particularly those with UPVD [26,27] and it is important to note that in the current study, patients with UPVD never reported any adverse effects during the performance of vestibular rehabilitation therapy using the BRU^TM^ system. Additional evidence is available on the efficacy of the BRU^TM^ system in older adults' vestibular rehabilitation, proving that it is an effective and recognized intervention for improving balance, boosting confidence, and preventing accidents in this population [30].

## Conclusions

The present research demonstrates the efficacy of the BRU^TM^ system of rehabilitation in patients with UPVD as compared to conventional vestibular rehabilitation therapy, which is essential for enhancing patient outcomes. Importantly, conventional vestibular rehabilitation is typically administered without medical supervision, which can result in improper exercise execution and therapy abandonment. In contrast, the BRU^TM^ system offers constant supervision throughout therapy administration. Comparing the BRU^TM^ technology to supervised conventional therapy revealed its efficacy and the benefits of using the BRU^TM^ system in motivating patients and supporting their confidence to improve treatment adherence and reduce symptoms. Contradictory evidence exists regarding the efficacy of the BRU^TM^ system and other VR devices in patients with UPVD and peripheral vestibular dysfunctions, emphasizing the need for further research to establish dosage parameters and provide specific recommendations for their use.
